# High Energetic Demand of Elite Rowing – Implications for Training and Nutrition

**DOI:** 10.3389/fphys.2022.829757

**Published:** 2022-04-19

**Authors:** Kay Winkert, Juergen M. Steinacker, Karsten Koehler, Gunnar Treff

**Affiliations:** ^1^ Division of Sports and Rehabilitation Medicine, Ulm University Medical Center, Ulm, Germany; ^2^ Department of Sport and Health Sciences, Technical University of Munich, Munich, Germany; ^3^ Institute of Sports Medicine, Prevention and Rehabilitation, Paracelsus Medical University, Salzburg, Austria

**Keywords:** elite sport, indirect calorimetry, exercise energy expenditure, rowers, nutrition

## Abstract

**Purpose:** Elite rowers have large body dimensions, a high metabolic capacity, and they realize high training loads. These factors suggest a high total energy requirement (TER), due to high exercise energy expenditure (EEE) and additional energetic needs. We aimed to study EEE and intensity related substrate utilization (SU) of elite rowers during rowing (EEE_ROW_) and other (EEE_NON-ROW_) training.

**Methods:** We obtained indirect calorimetry data during incremental (N = 174) and ramp test (N = 42) ergometer rowing in 14 elite open-class male rowers (body mass 91.8 kg, 95% CI [87.7, 95.9]). Then we calculated EEE_ROW_ and SU within a three-intensity-zone model. To estimate EEE_NON-ROW_, appropriate estimates of metabolic equivalents of task were applied. Based on these data, EEE, SU, and TER were approximated for prototypical high-volume, high-intensity, and tapering training weeks. Data are arithmetic mean and 95% confidence interval (95% CI).

**Results:** EEE_ROW_ for zone 1 to 3 ranged from 15.6 kcal·min^−1^, 95% CI [14.8, 16.3] to 49.8 kcal·min^−1^, 95% CI [48.1, 51.6], with carbohydrate utilization contributing from 46.4%, 95% CI [42.0, 50.8] to 100.0%, 95% CI [100.0, 100.0]. During a high-volume, a high-intensity, or a taper week, TER was estimated to 6,775 kcal·day^−1^, 95% CI [6,651, 6,898], 5,772 kcal·day^−1^, 95% CI [5,644, 5,900], or 4,626 kcal∙day^−1^, 95% CI [4,481, 4,771], respectively.

**Conclusion:** EEE in elite open-class male rowers is remarkably high already during zone 1 training and carbohydrates are dominantly utilized, indicating relatively high metabolic stress even during low intensity rowing training. In high-volume training weeks, TER is presumably at the upper end of the sustainable total energy expenditure. Periodized nutrition seems warranted for rowers to avoid low energy availability, which might negatively impact performance, training, and health.

## Introduction

Elite open-class male rowers are characterized by large body dimensions of about 193 cm standing height and 94 kg body mass (Kerr et al., 2007). They also have a high aerobic capacity or maximum oxygen consumptions (V̇O_2_max) of up to 6.9 L·min^−1^ (Nielsen and Christensen, 2020), as a consequence of their high blood volume and muscle mass (Treff et al., 2014), high percentages of oxidative muscle fibers (Roth et al., 1993; Maciejewski et al., 2020), and high cardiac output (Volianitis et al., 2020). This unique combination of high aerobic and endurance capacity and overall muscular strength allows elite rowers to generate approximately 892 W peak power per rowing stroke (Lawton et al., 2013). Mechanical power output averages at approximately 590 W during a rowing race (Steinacker, 1993) in which the athletes cover the 2,000-m distance in about 5.5–6.5 min. Such performance necessitates a considerable anaerobic contribution of 12–33% (Roth et al., 1983; Pripstein et al., 1999) and the ability to tolerate extreme metabolic acidosis with a pH as low as 6.74 (Nielsen, 1999). This energy demand stresses the metabolic pathways extremely and consequently Olympic rowing has been deemed the ultimate challenge to the human body (Volianitis and Secher, 2009).

To maximize performance and to prepare for racing, rowers train 15–30 h per week. Rowing clearly dominates their training routine, but unspecific endurance training, resistance training, and stretching complement the program (Fiskerstrand and Seiler, 2004; Tran et al., 2015). Training intensity distribution, commonly accessed by a three-zone model, has been reported to follow a pyramidal distribution, with ∼ 85% low intensity training, ∼ 12% threshold training, and ∼ 3% spent at high intensities (Plews et al., 2014; Treff et al., 2017). It is worth mentioning that training intensity distributions differ considerably among international rowing programs (Treff et al., 2021b) and shifts towards a polarized distribution during certain phases of the competition period have been reported (Treff et al., 2017).

Due to the volume and complexity of a rower’s training, dedicated planning and monitoring of the total training load is warranted. This necessitates an integrated approach of external and internal training load (Bourdon et al., 2017). While the external training load (e.g., training distance or duration) is generally well assessable (Treff et al., 2017), the quantification of internal load is far more challenging. Tools like the recovery stress questionnaire (Kellmann et al., 2001), measures of the autonomous nervous system (Plews et al., 2014), or biomedical markers (Hecksteden et al., 2016; Bizjak et al., 2021) that have been proposed to mirror acute or midterm stress, are frequently applied. While all these markers are surrogates for the organism’s response to repeated exercise, they fail to quantify the energetic load of training, which is on the cellular level the major stimulus connecting nutrient intake and training adaption (Hawley et al., 2018).

The energetic load of training is reflected by the energy expenditure. Messonnier and colleagues (Messonnier et al., 2005a) aimed to assess the training load in international open-class and lightweight rowers based on questionnaires. They reported a mean habitual weekly energy expenditure of 5,388 ± 159 kcal∙day^−1^. Others reported a nutrient intake of 7,000 kcal·day^−1^ in elite open-class male rowers during high-volume training (Boegman and Dziedzic, 2016).

To assess the total energy requirement (TER) of a rower precisely, non-exercise and exercise energy expenditure (EEE) need to be considered. EEE in elite open-class male rowers is presumably high, because of the large body dimensions, the high training volume, the involvement of approximately 70% active muscle mass during rowing (Steinacker, 1993), and the low mechanical efficiency of rowing of about 16–24% (Di Prampero et al., 1971). Taken altogether this suggests a very high EEE already at moderate training intensities, which represent the major part of the TER in elite rowers (Messonnier et al., 2005a). The non-exercise energy expenditure is more complex and thus difficult to predict. On the one hand, a high resting metabolic rate of 2,675 kcal∙day^−1^ has already been reported in open-class male rowers (Carlsohn et al., 2011), but this high energy demand is contrasted by a sedentary behavior outside of the daily training routine (i.e., off-training) (Sperlich et al., 2017). On the other hand, a more recent study reported 2.2 h∙week^−1^of moderate to vigorous off-activities above 60% of maximal heart rate (Treff et al., 2021a), which in turn already in itself corresponds to a rather active lifestyle.

In the light of the high EEE and possibly high TER, an energetic limitation of rowing training volume seems possible (Mader and Hollmann, 1977) and sufficient energy intake becomes a potential issue when training volume is high. Thus, more adequate data on EEE and TER in elite open-class male rowers are needed to adjust energy supply and expenditure, because otherwise risk of inadequate dietary energy intake is apparent (Braakhuis et al., 2013).

We therefore aimed to quantify EEE and substrate utilization of rowing training in elite open-class male rowers during laboratory-based ergometer testing, and aimed to approximate the accumulated TER for prototypical periodized training. To the best of our knowledge, this is the first study in elite open-class male rowers dedicated to this aim.

## Materials and Methods

### Overview

We retrospectively analyzed data from 174 incremental step and 42 incremental ramp tests conducted repeatedly in 14 elite open-class male rowers. All tests were part of the German Rowing federation’s testing routines between 2013 and 2020. Step tests were used to determine lactate thresholds, mechanical power output, heart rate (HR), oxygen uptake (V̇O_2_), carbon dioxide production (V̇CO_2_), and respiratory exchange ratio (RER) at each stage, while ramp tests were used to measure V̇O_2_max. Based on LTs and V̇O_2_max, the intensity continuum was divided into three zones adapting previous recommendations (Seiler, 2010; Manunzio et al., 2016).

EEE during ergometer rowing (EEE_ROW_) was accessed *via* indirect calorimetry, which also allowed for an estimation of the substrate utilization at a given workload. The percentage and absolute increase in carbohydrate metabolism, with a concomitant decrease in fatty acid utilization above moderate intensities (Brooks and Trimmer, 1996), is reflected by an increase in RER. Accordingly, RER allows for a specification of substrate utilization, thereby providing a further measure of (relative) exercise intensity in addition to percentage of V̇O_2_max, ventilatory, or lactate “threshold”.

For a given EEE, a corresponding amount of stored energy in the form of body fat and glycogen is required. We approximated the glycogen depletion associated with a given EEE_ROW_ based on body composition data and empirical data of liver and muscle glycogen, and blood glucose.

EEE for typical non-rowing sessions (EEE_NON-ROW_) was calculated based on metabolic equivalents of task (MET) corrected for resting metabolic rate of the appropriate sports mode (e.g., indoor cycling). Using EEE_ROW_ and EEE_NON-ROW_, we approximated daily TER for prototypical training weeks focusing either on high volume, high intensity, or on taper training.

### Participants

Fourteen elite open-class male rowers (body mass 91.8 kg, 95% confidence interval (95%CI) [87.7, 95.9], fat free mass 83.5 kg, 95% CI [80.1, 86.9], V̇O_2_max 6.6 L·min^−1^, 95% CI [6.5, 6.7] or 72.0 ml·min^−1^∙kg^−1^ [69.6, 74.5]) decorated with several medals from Olympic Games and/or World Championships participated in this study. Participants completed a varying number of tests during the observation period, depending on whether the participants continued to qualify for the national team and/or because of absences from tests for health or other reasons. All rowers were familiar with the pre-testing and testing procedures and maintained a balanced diet. They conducted no high-intensity training 48 h prior testing and the last low intensity training session ended 20 h before each test or earlier to avoid fatigue, glycogen deficiency, and hypohydration. The study was conducted according to the declaration of Helsinki and approved by the ethical board of the University of Ulm (##267/11). All participants gave written informed consent to participate in the testing and the retrospective data analyses.

### Equipment

All tests were conducted on a Concept 2 rowing ergometer (indoor rower, model D, Concept 2, Morrisville, United States). As described elsewhere (Mentz et al., 2020), the ergometer was equipped with a load cell and a rotary transducer to measure force and the travel distance of the handlebar, thereby allowing to calculate mechanical power output (Institut für Forschung und Entwicklung von Sportgeräten (FES), Berlin, Germany).

An automated metabolic analyzer equipped with a dynamic micro mixing chamber (Metamax 3x, Cortex Biophysics, Leipzig, Germany) measured V̇O_2_ and V̇CO_2_. Validity has been reported as 1.98 ± 2.98% difference to the Douglas bag method (Larsson et al., 2004). Blood lactate was measured using an amperometric-enzymatical analyzer (C-Line, EKF, Barleben, Germany). Reliability expressed as coefficient of variation (CV) of the device amounts to < 1.5% (Kohler and Boutellier, 2004). Calibration and maintenance of the ergometer setup and the metabolic and lactate analyzers were performed according to the manufacturers’ guidelines (EKF-diagnostic GmbH, 2013; Cortex Medical, 2015).

### Procedures and Testing

#### Determination of Lactate Thresholds

After a 30-min standardized warm-up at 150 W, all rowers performed a submaximum incremental test on the rowing ergometer. Steps lasted 4 min and increased by 50 W. Workload ranged 200–400 W in ten rowers or 200–450 W in five rowers with exceptionally high endurance performance, respectively. Gas exchange and ventilation were measured continuously and data were averaged over the last 30 s of each stage. During a 30-s break after each stage, 20 µL of capillary blood were drawn from the hyperemic earlobe and the concentration of capillary blood lactate was analyzed. Lactate “thresholds” 1 and 2 (LT1 and LT2) were calculated according to Dickhuth and colleagues (Dickhuth et al., 1991), using a polynomic fitting of the data (Winlactat, Mesics, Münster, Germany). Data of mechanical power output, ventilation, and gas exchange were aligned to LT1 and LT2, respectively.

#### Measurement of Maximum Oxygen Uptake

After a 30-min standardized warm-up at 150 W on a rowing ergometer, all rowers performed an incremental ramp test on the same rowing ergometer used for the incremental step tests. Gas exchange and ventilation were measured with the same metabolic analyzer applied in the step tests. The initial target power output was 160 W and increased by 30, 35, or 40 W·min^−1^, depending on the individual rower’s estimated performance level. The test was automatically terminated if a rower failed to increase mechanical power output within a 7-W range of five strokes (Treff et al., 2018). V̇O_2_max was defined as the highest 30-s moving average and considered as maximum if V̇O_2_ failed to increase with progressive work rate (leveling-off) or at least a plateau (i.e., increase in V̇O_2_ < 150 ml·min^−1^) was observed (Midgley et al., 2007).

### Calculations of Exercise Energy Expenditure, Glycogen Depletion, and Total Energy Requirement

#### Intensity-Zones

The metabolic load at LT1, 50% of LT2 (LT2_50%_) (Manunzio et al., 2016), LT2 and V̇O_2_max was applied to a three-zone model (Seiler, 2010), with zone 1 ranging from LT2_50%_ to LT1, zone 2 ranging from LT1 to LT2, and zone 3 ranging from LT2 to V̇O_2_max.

#### Calculation of EEE and SU for Rowing

EEE_ROW_ and substrate utilization at LT1, LT2_50%_, LT2, and V̇O_2_max were calculated using a non-protein table by Péronnet and colleagues (Péronnet and Massicotte, 1991). EEE_ROW_ was corrected by adding additional energy derived from the anaerobic energy contribution (EC_Lac_) and subtracting the resting metabolic rate. EC_Lac_ was derived by applying a O_2_-lactate equivalent according to Equation 1:

$${\mathrm{EC}}_{Lac}\;\left[kcal\right]=\;\Delta_{blood\;lactate}\left[mmol\cdot L^{-1}\right]\times 0.0033\;\;\left[L\cdot kg^{-1}\cdot \left(mmol\cdot L^{-1}\right){\;}^{-1}\right]\;\times \;BM\left[kg\right]\times 5.04\;\left[kcal\cdot L^{-1}\right]$$ (1)

Where Δ_blood_ lactate is the difference between rest and post-step or post-test blood lactate concentration, 1 mmol · L^−1^ Δ_blood_ lactate is equivalent to the energy released by the uptake of 0.0033 L of O_2_ per kg body mass (BM) (Margaria et al., 1963; Di Prampero and Ferretti, 1999), and 5.04 kcal represents the caloric equivalent of 1 liter O_2_. Resting metabolic rate was calculated according to Cunningham (Cunningham, 1980).

We calculated EEE_ROW_ and corresponding substrate utilization for typical steady-state sessions within the three intensity zones for different durations.

#### Calculation of EEE_NON-ROW_

We calculated EEE_NON-ROW_ for steady-state exercise at a given intensity and duration using estimated METs that were corrected for resting metabolic rate according to Equation 2 (Ainsworth et al., 2011):

$$EEE_{NON-ROW}\left[kcal\cdot min^{-1}\right]=\left(MET-1\right)\times RMR\;\left[kcal\cdot min^{-1}\right]$$ (2)

where MET values were taken from the Compendium of Physical Activities (Ainsworth et al., 2011) for cycling (compendium codes 1,050/1,040), strength training (2,050), calisthenics (2,030), stretching (2,101), and soccer (15,610). Again, resting metabolic rate (RMR) was calculated according to Cunningham (Cunningham, 1980).

#### Calculation of Glycogen Depletion

Glycogen depletion was calculated as a function of glycogen consumption (g·min^−1^) and duration of rowing training (minutes), for a given intensity zone based on the sum of carbohydrates from blood glucose, liver, and active skeletal muscle glycogen stores. Blood glucose mass was calculated based on plasma volume according to Equation 3:

$$Blood\;glucose\;\left[g\right]=\left(0.07\;\left[L\cdot kg^{-1}\right]\times LBM\;\left[kg\right]+0.06\left[L\right]\right)\times 1\;\left[g\cdot L^{-1}\right]$$ (3)

with the first term representing the plasma volume estimated from lean body mass (LBM) (data provided from Treff et al., 2014) and the second term (1 g·L^−1^). which is the upper limit of the normal glucose concentration in the blood plasma in the fasting state. LBM was determined *via* bioimpedance measurements (InBody 720, BioSpace, Seoul, Korea). Liver glycogen energy was calculated based on Equation 4:

$$Liver_{Energy}\;\left[kcal\right]=\left(BM\;\left[kg\right]\times 0.025\right)\times 195\;\left[kcal\cdot kg^{-1}\right]\;or\;365\;\left[kcal\cdot kg^{-1}\right]\;$$ (4)

with 0.025 corresponding to the proportion of liver *vs*. body mass (BM) (Valentin, 2002), assuming 2.3 kg, 95% [2.2; 2.4] liver mass and 195 kcal·kg^−1^ or 365 kcal·kg^−1^ representing the lower and upper end of liver glycogen density (Rapoport, 2010). For muscle glycogen, we determined an active skeletal muscle mass (SMM_Active_) of 33.7 kg, 95% [32.2, 35.2] *via* bioimpedance measurements and we assumed 70% active muscle mass in rowing (Steinacker, 1993). The lower and upper muscle glycogen content was further calculated according to Equation 5:

$$Muscle_{Glycogen}\;\left[g\right]=\left(SMM_{Active}\left[kg\right]\times 0.2\right)\;\times 0.18\;\left[g\cdot mmol^{-1}\right]\times 500\;\left[mmol\cdot kg^{-1}\right]\;or\;700\;\left[mmol\cdot kg^{-1}\right]$$ (5)

were 0.2 is the transformation factor from wet to dry weight (dw), 0.18 g·mmol^−1^ is the molecular weight of glucose, 500 mmol · kg^−1^ or 700 mmol · kg^−1^ are the assumed lower and upper limits of glycogen density per kg dw (Hearris et al., 2018).

To convert mass (g) of carbohydrates into energy (kcal), or vice versa, we applied the caloric equivalent of 4.1 kcal·g^−1^.

#### Calculation of Recommended Energy Availability and Total Energy Need

To account for the additional energy requirements of activities outside of EEE_ROW_ and EEE_NON-ROW_, we calculated the TER as the sum of EEE and the recommended energy availability (EA_REC_). The latter describes the amount of energy that is available for all other physical functions after subtracting the EEE from total energy expenditure (TEE) (Loucks, 2013) and was calculated according to Equation 6 (Areta et al., 2020):

$$EA_{REC}\left[kcal\cdot d^{-1}\right]=40\;\left[kcal\cdot kg^{-1}\cdot day^{-1}\right]\times FFM\;[kg]$$ (6)

where FFM depicts the fat free mass that was determined *via* bio impedance measurements. Based on these calculations, we approximated accumulated daily TER according to Equation 7:

$$TER\;\left[kcal\cdot d^{-1}\right]=AEE_{ROW}\;\left[kcal\cdot d^{-1}\right]+AEE_{NON-ROW}\;\left[kcal\cdot d^{-1}\right]+EA_{REC}\;\left[kcal\cdot d^{-1}\right]$$ (7)

#### Prototypical Training Sessions and Weeks

As a blueprint for typical training weeks we used original training plans of elite German rowers and calculated TER according to the previous equations. We selected exemplary high volume, high intensity, and tapering weeks. Training volume of these weeks amounted to 1,605, 1,055, and 555 min·week^−1^, respectively. Percentage of training spent in Zone 1–Zone 2–Zone 3 was 97.5%-2.5%-0.0% in the high-volume, 92.8%-0.0%-7.2% in the high-intensity, and 97.8%-0.0%-2.2% in the tapering week, respectively. Additional information is given in Table 1.

**TABLE 1:** Training indices for exemplary high volume, high intensity, and tapering rowing training weeks.

		Mo.	Tu.	We.	Th.	Fr.	Sa.	Su.	Sum
High volume	Sessions (N)	4 (2/2)	2 (1/1)	3 (2/1)	4 (2/2)	2 (1/1)	3 (2/1)	3 (2/1)	23 (13/10)
	(rowing/other)								
	Duration (min)	295 (190/105)	160 (100/60)	230 (170/60)	305 (200/105)	170 (90/80)	220 (160/60)	225 (180/45)	1,605 (1,090/515)
	(rowing/other)								
	TID (%) rowing	(100/0/0)	(100/0/0)	(100/0/0)	(100/0/0)	(70/30/0)	(100/0/0)	(100/0/0)	(97.5/2.5/0)
	(Z1/Z2/Z3)								
High intensity	Sessions (N)	3 (2/1)	3 (2/1)	2 (1/1)	3 (2/1)	2 (1/1)	3 (2/1)	2 (1/1)	18 (11/7)
	(rowing/other)								
	Duration (min)	190 (160/30)	170 (125/45)	140 (80/60)	170 (125/45)	140 (80/60)	170 (125/45)	75 (45/30)	1,055 (740/315)
	(rowing/other)								
	TID (%) rowing	(95/0/5)	(93.2/0/6.8)	(90/0/10)	(93.2/0/6.8)	(93/0/7)	(93.9/0/6.1)	(85/0/15)	(92.8/0/7.2)
	(Z1/Z2/Z3)								
Tapering	Sessions (N)	3 (2/1)	free	2 (2/0)	2 (1/1)	2 (2/0)	2 (1/1)	1 (1/0)	12 (9/3)
	(rowing/other)								
	Duration (min)	135 (105/30)	free	90 (90/0)	90 (60/30)	105 (105/0)	90 (60/30)	45 (45/0)	555 (465/90)
	(rowing/other)								
	TID (%) rowing	(97.4/0/2.6)	free	(100/0/0)	(95/0/5)	(97.7/0/2.3)	(100/0/0)	(95/0/5)	(97.8/0/2.2)
	(Z1/Z2/Z3)								

#### Statistical Analysis

To account for dependency of repeated step test (4–20 tests per rower) and ramp test (1-4 test per rower) measurements, unweighted individual mean values were calculated (Supplementary Table S1), with homogeneous individual standard deviations indicating no dependency of measurement variation on individual test repetitions. Consequently, individual mean values were further used to calculate robust descriptive data by the arithmetic mean with 95% CI. Descriptive data were calculated using SPSS (IBM Corp. Released 2017. IBM SPSS Statistics for Windows, Version 25.0. Armonk, NY: IBM Corp.).

## Results

Table 2 includes mechanical power output, cardiorespiratory, metabolic, and RPE data aligned to LT1, LT2, and V̇O_2_max.

**TABLE 2:** Mechanical, cardiorespiratory, metabolic demand and perceived rate of exhaustion [mean (95% CI)] for rowing at individual lactate thresholds and maximum oxygen consumption.

Variable	LT1	LT2	V̇O_2_max
Power (W)	274 [264, 284]	360 [348, 373]	520 [499, 540]
V̇O_2_ (L·min^−1^)	4.4 [4.2, 4.7]	5.5 [5.3, 5.7]	6.6 [6.5, 6.7]
%V̇O_2_max (%)	67.6 [64.2, 70.9]	83.6 [81.3, 85.8]	100.0 [100.0, 100.0]
RER ( )	0.90 [0.89, 0.91]	0.96 [0.96, 0.97]	1.07 [1.05, 1.09]
EEE (kcal·min^−1^)	21.3 [20.2, 22.4]	29.0 [28.0, 30.0]	48.3 [46.7, 49.9]
HR (min^−1^)	144 [140, 148]	168 [165, 171]	192 [187, 197]
Lac (mmol·L^−1^)	0.9 [0.8, 1.0]	2.4 [2.3, 2.5]	12.1 [11.4, 12.9]
RPE (a.u.)	3.0 [2.7, 3.3]	5.0 [5.0, 6.0]	—

Notes: Lactate threshold 1&2 (LT1&2) based on Dickhuth and colleagues (Dickhuth et al., 1991) based on incremental test. Maximum oxygen consumption data (V̇O_2_max) is based on ramp tests. V̇O_2_, oxygen consumption data; RER, respiratory exchange ratio; EEE, exercise energy expenditure; HR, heart rate; Lac, blood lactate concentration; RPE, rate of perceived exertion.

Table 3 includes mechanical power output, cardiorespiratory, and metabolic data with corresponding EEE_ROW_ and SU for a three-zone model based on LT1, LT2, and V̇O_2_max. EEE_ROW_ ranged from 15.6 kcal·min^−1^, 95% CI [14.8, 16.3] to 49.8 kcal·min^−1^, 95% CI [48.1, 51.6] and carbohydrate utilization ranged from 46.4%, 95% CI [42.0, 50.8] to 100%, 95% CI [100.0, 100.0], respectively.

**TABLE 3:** Mechanical, cardiorespiratory and metabolic demand of rowing corresponding to a three-zone model.

Variable	Zone 1		Zone 2		Zone 3	
	LL	UL	LL	UL	LL	UL
Power (W)	180 [174, 186]	274 [264, 284]	275 [265, 285]	360 [348, 373]	361 [349, 374]	520 [499, 540]
HR (min^−1^)	122 [117, 128]	144 [140, 148]	145 [141, 149]	168 [165, 171]	169 [166, 172]	192 [187, 197]
%HR_max_ (%)	63.7 [61.6, 65.7]	74.8 [73.4, 76.1]	75.3 [74.0, 76.7]	87.5 [86.1, 89.0]	88.1 [86.6, 89.5]	100.0 [100.0, 100.0]
V̇O_2_ (L·min^−1^)	3.4 [3.3, 3.6]	4.4 [4.2, 4.7]	4.4 [4.2, 4.7]	5.5 [5.3, 5.7]	5.5 [5.3, 5.7]	6.6 [6.5, 6.7]
%V̇O_2_max (%)	52.2 [49.8, 54.6]	67.6 [64.2, 70.9]	67.6 [64.3, 70.9]	83.6 [81.3, 85.8]	83.6 [81.4, 85.8]	100.0 [100.0, 100.0]
RER ( )	0.83 [0.82, 0.85]	0.9 [0.89, 0.91]	0.91 [0.90, 0.92]	0.96 [0.95, 0.97]	0.97 [0.96, 0.98]	1.07 [1.05, 1.09]
Lac (mmol·L^−1^)	0.7 [0.6, 0.7]	0.9 [0.8, 1.0]	1.0 [0.9, 1.1]	2.4 [2.3, 2.5]	2.5 [2.4, 2.6]	12.1 [11.4, 12.9]
EEE (kcal·min^−1^)	15.6 [14.8, 16.3]	21.3 [20.2, 22.5]	21.4 [20.2, 22.6]	29.2 [28.2, 30.2]	29.4 [28.4, 30.4]	49.8 [48.1, 51.6]
CHO (%)	46.4 [42.0, 50.8]	67.6 [64.4, 70.8]	67.6 [64.4, 70.8]	88.1 [85.7, 90.5]	88.4 [86.2, 90.6]	100.0 [100.0, 100.0]
LIP (%)	53.6 [49.2, 58.0]	32.4 [29.2, 35.6]	32.4 [29.2, 35.6]	11.9 [9.5, 14.3]	11.6 [9.4, 13.8]	0.0 [0.0, 0.0]
CHO (g·min^−1^)	1.8 [1.6, 2.0]	3.5 [3.2, 3.8]	3.6 [3.2, 3.9]	6.3 [6.0, 6.7]	6.4 [6.1, 6.7]	12.2 [11.7, 12.6]
LIP (g·min^−1^)	0.9 [0.8, 1.0]	0.7 [0.7, 0.8]	0.7 [0.7, 0.8]	0.3 [0.3, 0.4]	0.3 [0.3, 0.4]	0.0 [0.0, 0.0]

Notes: Data are arithmetic means and [95% confidence interval] within a three-zone model with lower limits (LL) and upper limits (UL) based on zone 1 LT2_50%_–LT1, Zone 2 LT1–LT2, Zone 3 LT2 - maximum oxygen consumption (V̇O_2_max). Calculation of exercise energy expenditure (EEE) and substrate utilization in carbohydrates (CHO) and lipids (LIP) based on a non-protein table (Péronnet and Massicotte, 1991) and corrected for resting metabolic rate (Cunningham, 1980) and anaerobic energy contribution (Di Prampero and Ferretti, 1999). HR, heart rate; V̇O_2_, oxygen consumption; RER, respiratory exchange ratio; Lac, blood lactate concentration.

**FIGURE 1:**
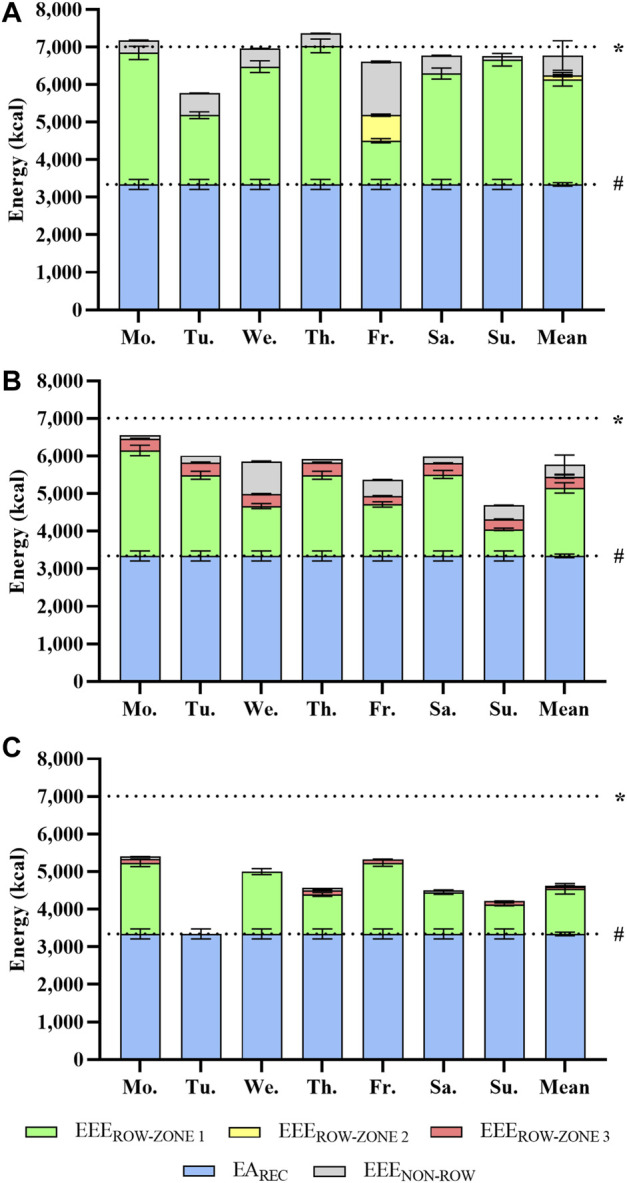
Total energy requirement by exercise energy expenditure and recommended energy availability (EA_REC_) for an exemplary high volume **(A)**, high intensity **(B)**, and tapering rowing training week **(C)** (mean [95% confidence interval]). Calculation of exercise energy expenditure for rowing (EEE_ROW_) training based on a non-protein table (Péronnet and Massicotte, 1991) and corrected for resting metabolic rate (RMR) and anaerobic energy contribution (Di Prampero and Ferretti, 1999). EEE for other training (EEE_NON-ROW_) is approximated using corrected MET data (Kozey et al., 2010). EA_REC_ (dashed line #) is given as 40 kcal·kg^−1^ fat free mass·day^−1^ (Koehler et al., 2016). A total energy expenditure of three times the RMR (7,011 kcal·day^−1^, dashed line *) was assumed to reflect the upper limit of the manageable total energy expenditure (Thurber et al., 2019).

Figure 1 shows the accumulated daily mean EEE, separated for EEE_ROW_ in zones 1-3 and EEE_NON-ROW_ for each of the prototypical training weeks. Descriptive training data for these weeks are shown in Table 1. The estimated mean weekly EEE_ROW_ during a high volume, high intensity, or tapering week amounted to 2,899 kcal∙day^−1^, 95% CI [2,749, 3,049], 2,110 kcal∙day^−1^, 95% CI [1,970, 2,251], or 1,257 kcal∙day^−1^, 95% CI [1,157, 1,356]. EEE_NON-ROW_ was 533 kcal∙day^−1^, 95% CI [455, 612], 319 kcal∙day^−1^, 95% CI [268, 371], or 27 kcal∙day^−1^, 95% CI [27, 27], respectively. Summarized with an estimated EA_REC_ of 3,343 kcal∙day^−1^, 95% CI [3,211, 3,474], mean weekly TER amounted to 6,775 kcal·day^−1^, 95% CI [6,651, 6,898], 5,772 kcal·day^−1^, 95% CI [5,644, 5,900] or 4,626 kcal∙day^−1^, 95% CI [4,481, 4,771] for these prototypical weeks.

**FIGURE 2:**
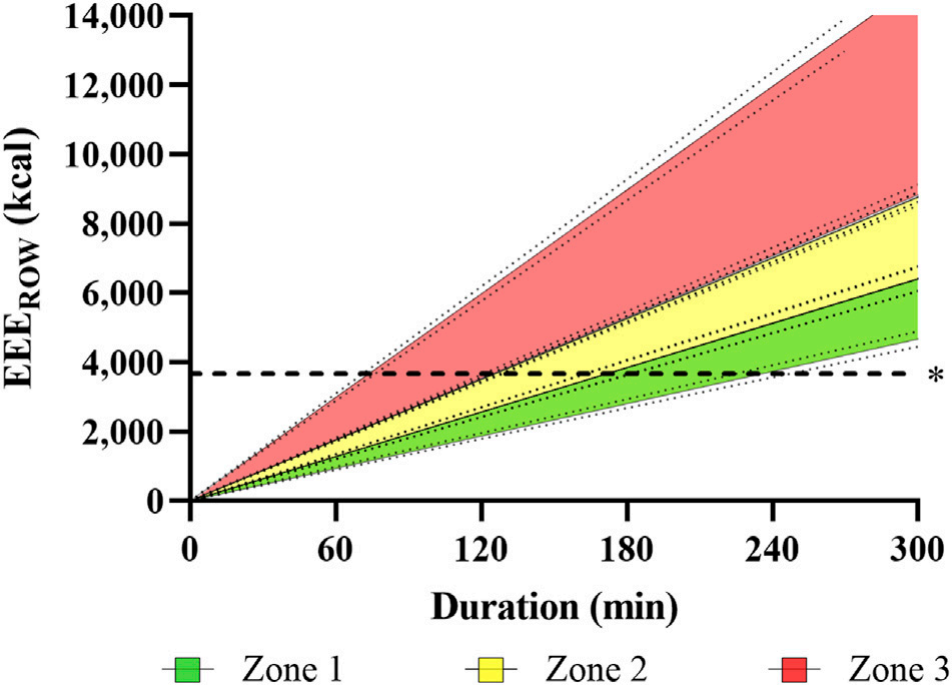
Illustration of estimated, accumulated exercise energy expenditure over time during rowing (EEE_ROW_) for a three-zone model, based on indirect calorimetry data obtained in incremental step testing. The Figure visualizes accumulated EEE for each training zone over time, assuming a linear progression with 95% confidence intervals. *R* ^2^ ranged 0.968–0.988. Dashed line represents EEE of 3,668 kcal·day^−1^, 95% CI [3,580, 3,756], which is assumably the maximum refuelable EEE.

Figure 2 illustrates the accumulated EEE_ROW_ as a function of rowing training duration for each intensity zone.

**FIGURE 3:**
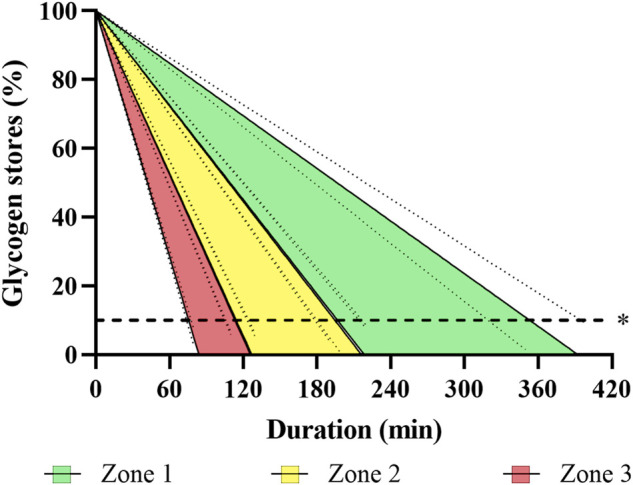
Illustration of estimated glycogen depletion over time during rowing for a three-zone model, based on indirect calorimetry data obtained in incremental step testing. The Figure visualizes depletion of individually estimated glycogen stores (including blood, liver, and muscle; see text for details) over time, assuming a linear progression with 95% confidence intervals. *R* ^2^ ranges 0.781–0.985. *Dashed line represents the 90% maximum depletion threshold of glycogen stores (Burke et al., 2017).

Figure 3 illustrates the muscle glycogen depletion as a function of the intensity zones, utilization rate of carbohydrates, and duration of rowing training. Approximated blood glucose, liver, and muscle glycogen stores amounted to 6 g, 95% CI [5, 6], 109 g, 95% CI (104, 114) to 201 g, 95% CI (193, 210), and 607 g, 95% CI [580, 634] to 850 g, 95% CI [812, 888], respectively. In total, this corresponded to 2,959 kcal, 95% CI [2,828, 3,091] to 4,333 kcal, 95% CI [4,141, 4,526].

## Discussion

We studied EEE and substrate utilization of ergometer rowing in elite male open-class rowers. Indirect calorimetry data were obtained during repeated incremental step and ramp testing to calculate EEE_ROW_ and substrate utilization in three intensity zones. Further, we approximated daily TER as the sum of EEE_ROW_, EEE_NON-ROW_, and EA_REC_ for prototypical high volume, high intensity, and tapering training weeks.

The main results are a high EEE_ROW_ in elite open-class male rowers ranging 15.6 kcal·min^−1^, 95% CI [14.8, 16.3] to 48.8 kcal·min^−1^, 95% CI [48.1, 51.6] from zone 1 to 3. The energetic contribution of fat metabolism was never higher than 53.6 ± 7.6%. Furthermore, we calculated a high TER ranging 4,626 kcal∙day^−1^, 95% CI [4,481, 4,771] to 6,775 kcal·day^−1^, 95% CI [6,651, 6,898] during prototypical training weeks. This indicates that isocaloric energy intake may become challenging during high volume training weeks and points to a metabolic limitation of reasonable rowing training volume.

### Metabolic Demand of Ergometer Rowing

The metabolic demand within ergometer rowing is already high at LT1 and LT2. The mechanical power output at these “thresholds” (Table 2) corresponds well with previous data published for elite male open-class rowers (Tran et al., 2015). However, for the first time, we report V̇O_2_- and RER-data aligned to these points as well as to V̇O_2_max. While the high metabolic demand at V̇O_2_max is apparently related to the remarkably high absolute aerobic capacity of elite male open-class rowers, sub-maximum V̇O_2_ data at LT1 and LT2 are far more interesting. Here, the metabolic demand is considerably higher than corresponding results for e.g., cyclists, ranging 3.6–4.3 L·min^−1^ (Garvican et al., 2013). This is attributable to the relatively large body dimensions of rowers, especially their high body and muscle mass. Nevertheless, also a particularly of ergometer rowing — or more exactly its measurement — contributes to this phenomenon: During the drive phase, when the rower pushes off from the foot stretcher with his legs and moves backwards on his sliding seat, the rower exerts force on the handle or oar and this force is measured. Afterwards, the rower has to bring himself back to the starting position, which also requires force and energy. This additional mechanical power output—amounting to roughly 38 W (Mentz et al., 2021)—is not measured by common ergometers. Consequently, the actual mechanical power output is underestimated and the metabolic load of ergometer rowing *vs.* cycling at a given (displayed power output is considerably higher (Turner and Rice, 2021). Thus, the mechanical efficiency is significantly lower in ergometer rowing *vs*. cycling (Lindenthaler et al., 2018). Nevertheless, it is worth mentioning that ergometer rowing has been reported to induce similar metabolic stress as on-water rowing (Vogler et al., 2010). Another factor that contributes to the high V̇O_2_ data at LT1, LT2, and V̇O_2_max during rowing is the high amount of muscle mass recruited and the high venous return due to the sitting position of the rower (Volianitis and Secher, 2009), thereby resulting in a relatively high EEE at any given mechanical power output.

### Metabolic Demand of Ergometer Rowing at Low, Moderate, and High Intensities

The V̇O_2_max percentages derived from the lactate thresholds in our study fit well to previous categorizations (Seiler, 2010), underlining the validity of our data that allowed us to estimate the metabolic demand within each zone of the three-zone model (Table 2). EEE_ROW_ in all intensity zones was apparently high, ranging from 15.6 kcal·min^−1^, 95% [14.8.16.3] to 49.8 kcal·min^−1^, 95% [48.1, 51.6], equaling to an 10- to 31-fold increase in RMR, respectively. It seems worth to highlight that a “basic” endurance training in an elite open-class male rower at the upper range of zone 1 around LT1 already necessitates a V̇O_2_ of 4.4 L·min^−1^ something that is simply not possible for e.g., an elite distance runner with a V̇O_2_max of 4.2 L·min^−1^ (Jones et al., 2021). This illustrates the energetic differences between these elite athletes of different endurance sports disciplines.

However, aside from EEE, substrate utilization is of particular importance to rate the metabolic stress of exercise. While we estimate lipid stores in this sample of elite open-class male rowers as high as ∼ 77,000 kcal (9.3 kcal·g^−1^ x 8,300 g body fat mass), maximum energy stores from glycogen and blood glucose are limited to 2,959 kcal, 95% CI [2,828, 3,091] to 4,333 kcal, 95% CI [4,141, 4,526] with almost perfectly filled glycogen stores. Since glycogen stores largely depend on the loading status and assuming that no more than 90% depletion of the initial muscle glycogen store are tolerable (Burke et al., 2017), the lower value seems to reflect a more realistic scenario.

Our data indicate that energy during ergometer rowing training is mainly derived from carbohydrates, because even during a zone 1 training, about 47–68% of the energy originates from carbohydrates equaling 1.8 g·min^−1^, 95% CI [1.6, 2.0] to 3.5 g·min^−1^, 95% CI [3.2, 3.8]. Leaving aside other factors of fatigue or refueling, available energy stores from glycogen and blood glucose in this sample of elite rowers will theoretically allow for about 5.7 h, 95% CI [5.1, 6.3] to 3.1 h, 95% CI [2.8, 3.4] training in zone 1. In zone 2 and 3 theoretical maximum duration will be reduced by 47–74% (3.1 h, 95% CI [2.8, 3.4] to 1.8 h, 95% CI [1.6, 1.9]) and 70–86% (1.8 h, 95% CI [1.6, 1.9] to 1.0 [0.8, 1.0]), respectively. Again, due to other factors of fatigue and the unlikelihood of perfectly filled glycogen stores, the lower values are more realistic for common training scenarios.

Especially long-distance runners and cyclists try to maximize the fat oxidation rate through very low training intensities (Jeukendrup and Achten, 2001). Due to the high utilization of CHO observed in our rowers, such a “FatMax” training is apparently unlikely to be realized even during low zone 1 rowing. In line with this notion, Dandanell and colleagues (Dandanell et al., 2018) reported FatMax at 46% of V̇O_2_max in highly trained cross-country skiers. That is about 6% lower than the bottom of zone 1 training in our study (Table 3). While beyond the scope of this study, it seems worth to evaluate if such low intensities are warranted to prepare for high-intensity rowing races lasting not more than 7 min (Messonnier et al., 2005b).

### Metabolic Demand of Rowing Training Sessions

Based on the metabolic demand at each intensity zone, we calculated the EEE_ROW_ and substrate utilization for prototypical rowing training sessions and weeks, assuming that the metabolic demand of ergometer rowing generally reflects on water rowing (Vogler et al., 2010). Due to the high metabolic demand, a long basic endurance session lasting 100 min constantly rowed in zone 1 will result in an EEE_ROW_ ranging from 1,556 kcal, 95% CI [1,479, 1,670] to 2,133 kcal, 95% CI [2,019, 2,300]. Notably, such rowing sessions — at least in German rowing — are usually not performed at intensities considerably lower than LT1, because this would not allow to row the boat with the targeted propulsion and speed, corresponding to 72–80% of a boat’s world best time (Schwarzrock et al., 2017). Hence, the upper limit of the range is more likely to reflect the EEE_ROW_ of a realistic, 100-min basic endurance rowing session.

During such a 100-min zone 1 rowing session, the carbohydrate stores (muscle and liver glycogen as well as blood glucose) of the rowers will deplete with a degradation rate of 2.2 g·min^−1^, 95% CI [2.0, 2.4] to 3.9 g·min^−1^, 95% CI [3.6, 4.2], when accounting for EEE and RMR. This corresponds to a reduction to 75%, 95% CI [72, 77] to 54%, 95% CI [50, 59] of initial values (Figure 3)—with the latter being more realistic, as discussed before. Similar results were reported by Stepto and colleagues (Stepto et al., 2001) for aerobic interval training in competitive cyclists with muscle glycogen depletion ranging from 36 to 64%. Hence, not only the muscular impact of long rowing endurance sessions is demanding - they also include the risk of glycogen depletion. Consequently, sufficient carbohydrate ingestion during such exercises seems crucial to maintain glucose availability and pace. Importantly, in case of insufficient refueling, low pre-exercise muscle glycogen concentrations will not only result in reduced high-intensity performance (Maughan and Poole, 1981), but might also compromise immune function and training readiness (Stellingwerff et al., 2011).

### Metabolic Demand During Prototypical Training Weeks

Elite rowers regularly perform 2-4 sessions a day and they usually train for at least 6 days a week (Tran et al., 2015). To estimate the TER, we added the accumulated EEE of EEE_ROW_ and EEE_NON-ROW_ to the resting metabolic rate and EA_REC_. As shown in Figure 1 this leads to an energy demand that increases from tapering week, to high-intensity week, and finally peaks in the high-volume week with 6,778 kcal·day^−1^, 95% CI [6,651, 6,905]. If we assume that zone 1 training is in fact performed at the upper end of its intensity range, TER for the high-volume week would be even higher, and amount to 7,231 kcal·days^-1^, 95% CI [7,082, 7,380]. This is three times the resting metabolic rate of 2,337 kcal·day^-1^, 95% CI [2,262, 2,411].

While these TER values are very high, results from other sports underline their plausibility. Aside from extreme values of 9,869 ± 4,129 or 11,246 ± 1,083 kcal·day^−1^ reported during ultra-endurance competitions (Heydenreich et al., 2017; Geesmann et al., 2014), high caloric needs were also reported for elite male cross-country skiers. Sjödin and colleagues (Sjödin et al., 1994), for example, determined TEE *via* double labelled water during a 6-days training camp with an average training volume of 212 min·day^−1^ to be as high as 7,213 ± 1,003 kcal·day^−1^, notably with a slightly negative energy balance of -0.6%. These data fit well to TER estimated in our elite open-class male rowers. The “manageable” TEE during daily training conditions by resupplied energy through nutrition can be assumed to be three times the RMR (Thurber et al., 2019), amounting to 7,011 kcal·day^−1^, 95% CI [6,787, 7,234] in our elite open-class rowers (Figure 1). Assuming an EA_REC_ of 3,343 kcal·day^−1^, 95% CI [3,207, 3,479], about 3,668 kcal·day^−1^, 95% CI [3,580, 3,756] will remain for EEE (Figure 2). Hence, it becomes apparent that high-volume training of 1,605 min·week^−1^ in elite open-class male rowers is at the upper end of what is energetically possible or–more precisely–sustainable. Moreover, insufficient dietary energy intake with suppressed energy availability can disrupt different aspects of homeostasis in humans, a topic which is discussed as *relative energy deficiency in sport* (RED-S) (Mountjoy et al., 2018) and the *female athlete triad* (De Souza et al., 2014; De Souza et al., 2019a; De Souza et al., 2019b), both providing theoretical frameworks for physiological dysregulations and adverse effects on training capacity, performance, and health.

Considering the high EEE_ROW_ and the suspected need to exercise with relatively high intensities in order to provide an effective stimulus for improvements of already highly trained athletes (Treff et al., 2021a), it becomes clear that the capacity of elite athletes to complete high training volume is relatively lower than in untrained persons. The untrained can exercise with a lower absolute intensity, therefore need less energy and yet profit more from low metabolic demands. This dilemma highlights the limiting role of a high TEE in elite sports.

Practically, it is worth to consider that the differences in TER calculated for the three exemplary training weeks require an adjustment of energy intake. The latter is known as *periodized nutrition* (Jeukendrup, 2017). To account for fluctuations in training load, periodized nutrition necessitates at least an approximated TER. Thereby, estimates of an adequate reduction in energy intake from a high intensity to a tapering week can prevent unwarranted body mass gain. Vice versa, periodized nutrition based on valid data can help to prevent energy deficiency or RED-S (Mountjoy et al., 2018).

Our data also indicate that availability and recovery of glycogen stores may be a key factor for successful rowing training. With a mean rate of muscle glycogen synthesis ranging 5–6 mmol·kg^−1^·h^−1^ of wet muscle mass, normalization of muscle glycogen levels after extreme depletion will require 20–24 h (Coyle, 1991). Consequently, the calculated depletion to 75–54% of initial glycogen and blood glucose energy stores after 100 min zone 1 rowing training is unlikely to be sufficiently restored between daily training sessions. Especially during high volume training camps with repeated sessions on consecutive days (Table 3), athletes may not be able to fully restore muscle glycogen. Hence, glycogen concentration in active muscle will fall progressively over the day or a period of days. This may accidentally lead to a situation where glycogen stores are low in the second or third daily training session, where the achievable metabolic rate and exercise capacity are limited, because fat and protein metabolism are augmented. This is — in the end—an extended catabolic situation.

### Practical Implications

Due to the dominant contribution of carbohydrates during basic endurance training, their availability through restoration of glycogen stores and immediate provision is warranted during low-intensity rowing training lasting longer than 60 min. Of note, and in contrast to e.g., cyclists or triathletes (Sareban et al., 2016), such procedures seem to be not very common in the rowing culture, at least according to our own observations. Daily recommendation for the macronutrients range from 6–12 g carbohydrates ·kg^−1^ body mass·day^−1^, 1.5–1.7 g protein·kg^−1^ body mass·day^−1^, and 0.8–2.0 g fat·kg^−1^ body mass·day^−1^ (Stellingwerf et al., 2011). If athletes fail repeatedly to consume the recommended amounts of dietary carbohydrates (Baranauskas et al., 2015), the EEE of rowing may become a limiting factor. We recommend to consider this when planning training and nutrition for rowers. Otherwise, EEE and TER might surpass the maximum energy intake leading to potentially adverse effects for both health and performance.

### Limitations

As the intensity zones were calculated based on lactate "thresholds," we want to underline that we do not uncritically use the threshold term, and that we are aware of the undeniable changes associated with its conceptual basis (Poole et al., 2021). However, LT1 and LT2 still provide useful key measures for exercise description and definition of intensity zones.

We approximated EEE_ROW_ based on repeated ergometer tests, EEE_NON-ROW_ *via* corrected METs and we calculated EA_REC_. Due to the assumptions underlying such calculations, uncertainties are inherent. However, the repeated measurements in elite athletes during highly standardized laboratory conditions provide a previously unseen data quality. The transfer of ergometer rowing to on-water rowing may also be deemed a limitation, however Vogler and colleagues (Vogler et al., 2010) reported strong correlations between ergometer *vs*. on-water based incremental tests (blood lactate concentration r = 0.84, V̇O_2_ r = 0.91) and trivial to small differences of metabolic variables (blood lactate concentration −4.4% to 23.1%, V̇O_2_ −1.1% to −1.2%). Nevertheless, further individual validation seems warranted.

Notably, we simplified our calculations, assuming continuous, even paced steady-state training sessions with constant EEE and substrate utilization, neglecting that real-life training sessions are often characterized by changing pace, cardiovascular drift, and slow component kinetics in V̇O_2_ for intensities above LT1, surely causing fluctuations in EEE and substrate utilization. Finally, it was shown that the percentage of training spent below zone 1 may be as high as 30% of the total training time (Treff et al., 2021a), thereby suggesting another real-life scenario we did not account for. Though including the lactate equivalent, we did not precisely account for the oxidation of lactate, which spares glucose and glycogen during intense exercise (Miller et al., 2002) and we did not include the impact of lactate as a gluconeogenic precursor, as we are not aware of any data for sufficiently precise quantification that may be applied for our context.

Although our projections of EEE, TER, and substrate utilization during different training scenarios were made on the basis of highly unique data set of laboratory data designed to closely monitor real-life scenarios in elite athletes and include well-established literature references, external validation is warranted. The current gold standard for quantifying TEE that can be implemented in free-living individuals with minimal subject burden is the doubly labelled water method (Ainslie et al., 2003). In contrast, techniques to measure substrate utilization and specifically glycogen depletion are to this date highly invasive and require repeated collection of muscle biopsies (Hearris et al., 2018) and highly specialized imaging equipment and training (van Zijl et al., 2007). Future studies in elite rowers should aim to include these measures to confirm our predictions.

## Conclusion

Due to their large body dimensions and high metabolic capacity with high V̇O_2max_, the outstanding metabolic demand of rowing, and high training loads, the energy expenditure of elite open-class male rowers is extraordinarily high. At least in high volume weeks a metabolic limitation is likely, as the energy need is at the upper range of the maximum daily nutrient intake.

Importantly, the percentage of carbohydrates oxidized during slow long-distance rowing training is always dominant and rowing training mainly utilizing fat metabolism seems unrealistic. A single training session is suspected to cause a relevant glycogen depletion. Therefore, we recommend the consumption of carbohydrates during long rowing session and a systematic refilling between daily sessions. These notions should also be considered when planning training volume at a given intensity and the timing of individual sessions, because otherwise hypocaloric conditions and adverse effects are likely.

## Data Availability

The raw data supporting the conclusions of this article will be made available by the authors, without undue reservation.

## Abbreviations

EEE, Exercise energy expenditure; EEE_ROW_, Exercise energy expenditure during rowing; EEE_NON-ROW_, Exercise energy expenditure during training other than rowing; EA_REC_, Recommended energy availability; MET, Metabolic equivalent of task; TEE, Total energy expenditure; TER, Total energy requirement.
