# Supplementary material for: High Energetic Demand of Elite Rowing – Implications for Training and Nutrition
Source: Front Physiol. 2022 Apr 19;13:829757. doi: 10.3389/fphys.2022.829757 (PMC9062098; doi:10.3389/fphys.2022.829757)
Supplement: Supplementary file 1 [file Table1.DOCX]

Supplemental material: Table S1

Mechanical and cardiorespiratory demand (mean ±SD) at individual lactate thresholds and maximum oxygen consumption.

|  |  |  | **LT1** | | | |  | **LT2** | | | |  | **V̇O_2_max** | | | |
| --- | --- | --- | --- | --- | --- | --- | --- | --- | --- | --- | --- | --- | --- | --- | --- | --- |
| Rower | Step tests (n) | Ramp tests (n) | Power (W) | Lac (mmol·L^-1^) | V̇O_2_ (L·min^-1^) | RER ( ) |  | Power (W) | Lac (mmol·L^-1^) | V̇O_2_ (L·min^-1^) | RER ( ) |  | Power (W) | Lac (mmol·L^-1^) | V̇O_2_ L·min^-1^) | RER ( ) |
| 1 | 14 | 3 | 255 ±6 | 0.9 ±0.2 | 4.4 ±0.2 | 0.91 ±0.03 |  | 344 ±10 | 2.4 ±0.2 | 5.4 ±0.2 | 0.96 ±0.03 |  | 548 ±8 | 12.0 ±0.4 | 6.6 ±0.1 | 1.10 ±0.00 |
| 2 | 4 | 1 | 269 ±3 | 1.0 ±0.1 | 4.5 ±0.2 | 0.91 ±0.08 |  | 386 ±0 | 2.5 ±0.1 | 5.5 ±0.5 | 0.97 ±0.07 |  | 455 ±0 | 11.4 ±0.0 | 6.4 ±0.0 | 1.10 ±0.00 |
| 3 | 11 | 2 | 313 ±10 | 1.2 ±0.2 | 5.5 ±0.3 | 0.91 ±0.02 |  | 406 ±11 | 2.7 ±0.2 | 6.2 ±0.3 | 0.98 ±0.03 |  | 510 ±29 | 11.7 ±0.4 | 6.7 ±0.3 | 1.10 ±0.00 |
| 4 | 20 | 4 | 256 ±10 | 0.8 ±0.2 | 3.9 ±0.2 | 0.87 ±0.03 |  | 338 ±12 | 2.3 ±0.2 | 5.2 ±0.3 | 0.94 ±0.03 |  | 521 ±26 | 12.7 ±0.5 | 6.8 ±0.1 | 1.07 ±0.02 |
| 5 | 12 | 3 | 281 ±10 | 0.9 ±0.2 | 4.6 ±0.3 | 0.88 ±0.03 |  | 369 ±13 | 2.4 ±0.2 | 5.8 ±0.3 | 0.97 ±0.03 |  | 507 ±18 | 9.1 ±0.6 | 6.7 ±0.1 | 1.07 ±0.01 |
| 6 | 15 | 4 | 277 ±12 | 0.8 ±0.1 | 4.5 ±0.2 | 0.92 ±0.02 |  | 343 ±10 | 2.3 ±0.1 | 5.4 ±0.2 | 0.97 ±0.02 |  | 489 ±32 | 12.6 ±0.6 | 6.3 ±0.1 | 1.10 ±0.05 |
| 7 | 4 | 1 | 265 ±15 | 0.8 ±0.0 | 4.1 ±0.3 | 0.86 ±0.02 |  | 359 ±8 | 2.3 ±0.0 | 5.1 ±0.3 | 0.95 ±0.01 |  | 538 ±0 | 13.4 ±0.0 | 6.7 ±0.0 | 1.08 ±0.00 |
| 8 | 19 | 4 | 269 ±8 | 0.8 ±0.2 | 4.4 ±0.2 | 0.89 ±0.03 |  | 354 ±11 | 2.3 ±0.2 | 5.5 ±0.2 | 0.95 ±0.03 |  | 547 ±4 | 12.4 ±0.8 | 6.8 ±0.1 | 1.07 ±0.05 |
| 9 | 16 | 4 | 271 ±8 | 0.9 ±0.3 | 3.9 ±0.3 | 0.91 ±0.02 |  | 352 ±12 | 2.4 ±0.3 | 5.2 ±0.3 | 0.98 ±0.02 |  | 495 ±34 | 11.7 ±1.0 | 6.5 ±0.2 | 1.07 ±0.04 |
| 10 | 16 | 4 | 250 ±4 | 0.8 ±0.2 | 4.6 ±0.2 | 0.90 ±0.02 |  | 332 ±12 | 2.3 ±0.2 | 5.5 ±0.3 | 0.96 ±0.02 |  | 508 ±31 | 12.2 ±1.3 | 6.4 ±0.2 | 1.08 ±0.02 |
| 11 | 7 | 2 | 290 ±13 | 0.8 ±0.2 | 4.4 ±0.2 | 0.89 ±0.04 |  | 387 ±10 | 2.3 ±0.2 | 5.5 ±0.2 | 0.95 ±0.04 |  | 533 ±25 | 11.2 ±1.4 | 6.5 ±0.1 | 1.08 ±0.04 |
| 12 | 17 | 4 | 299 ±10 | 1.2 ±0.2 | 4.7 ±0.2 | 0.90 ±0.04 |  | 394 ±10 | 2.7 ±0.2 | 5.8 ±0.2 | 0.97 ±0.03 |  | 536 ±12 | 12.9 ±1.1 | 6.7 ±0.1 | 1.04 ±0.02 |
| 13 | 9 | 3 | 280 ±14 | 0.8 ±0.2 | 4.3 ±0.3 | 0.91 ±0.03 |  | 364 ±12 | 2.4 ±0.3 | 5.5 ±0.3 | 0.97 ±0.03 |  | 537 ±8 | 11.8 ±1.2 | 6.8 ±0.0 | 1.03 ±0.02 |
| 14 | 10 | 3 | 279 ±5 | 0.9 ±0.1 | 4.3 ±0.2 | 0.90 ±0.02 |  | 374 ±8 | 2.4 ±0.2 | 5.6 ±0.4 | 0.95 ±0.03 |  | 516 ±17 | 13.7 ±0.8 | 6.7 ±0.0 | 1.08 ±0.03 |
| Mean | 12 ±5 | 3 ±1 | 274 ±20 | 0.9 ±0.2 | 4.4 ±0.4 | 0.90 ±0.03 |  | 360 ±25 | 2.4 ±0.2 | 5.5 ±0.4 | 0.96 ±0.03 |  | 520 ±28 | 12.1 ±1.3 | 6.6 ±0.2 | 1.07 ±0.03 |

Notes: Lactate threshold 1 & 2 (LT 1 & 2) based on Dickhuth et al. 1991 (Dickhuth et al. 1991) based incremental test results. Maximum oxygen consumption (V̇O_2_max) based on ramp tests. Lac, blood lactate concentration; V̇O_2_, oxygen consumption; RER, respiratory exchange ratio.
